# Revisiting the JOQUER trial: stratification of primary Sjögren’s syndrome and the clinical and interferon response to hydroxychloroquine

**DOI:** 10.1007/s00296-021-04927-y

**Published:** 2021-06-24

**Authors:** Alexis Collins, Dennis Lendrem, James Wason, Jessica Tarn, Nadia Howard-Tripp, Iris Bodewes, Marjan A. Versnel, Jacques-Eric Gottenberg, Raphaele Seror, Xavier Mariette, Wan-Fai Ng

**Affiliations:** 1 Musculoskeletal Research Group, Translational and Clinical Research Institute, Newcastle University, Newcastle upon Tyne, UK; 2 NIHR Newcastle Biomedical Research Centre, Newcastle University and Newcastle Upon Tyne NHS Foundation Trust, Tyne and Wear, Newcastle upon Tyne, UK; 3 Faculty of Medical Sciences, Population Health Sciences Institute, Newcastle University, Newcastle upon Tyne, UK; 4 Department of Immunology, Erasmus University Medical Centre, Rotterdam, The Netherlands; 5 Department of Rheumatology, National Reference Centre for Rare Systemic Autoimmune Diseases, Strasbourg University Hospital, CNRS, IBMC, Université de Strasbourg, UPR 3572, Strasbourg, France; 6 Centre for Immunology of Viral Infections and Autoimmune Diseases, INSERM, UMR 1184, Université Paris- Saclay, Paris, France; 7 Department of Rheumatology, Assistance Publique-Hospitaux de Paris (APHP), Hôpital Bicêtre, Le Kremlin-Bicêtre, Paris, France

**Keywords:** Sjögren’s syndrome, Hydroxychloroquine, Interferon

## Abstract

To re-analyse the clinical outcomes and interferon (IFN) activity data from the JOQUER trial, a phase III trial investigating hydroxychloroquine (HCQ) in patients with primary Sjögren’s syndrome (pSS), after stratifying patients into putative pathobiological subgroups utilizing the Newcastle Sjögren’s Stratification Tool (NSST) based on patient-reported symptoms of dryness, pain, fatigue, anxiety and depression. 107 patients were assigned to one of four subgroups using NSST at baseline—the high symptom burden (HSB), pain dominant with fatigue (PDF), dryness dominant with fatigue (DDF) and low symptom burden (LSB). Endpoints were re-analysed after stratification, testing for treatment differences within subgroups and adjusting for baseline differences using a repeated measures covariate model. The HSB subgroup (*n* = 32) showed a relative improvement in ESSPRI of 1.49 points (95% CI 0.54–2.43; *p* = 0.002) within 12 weeks in patients taking HCQ compared to placebo, with no further changes after 24 weeks. For the LSB subgroup (*n* = 14), the ESSPRI worsened in the placebo but not the HCQ arm after 12 weeks (mean difference 1.44, 95% CI 0.05–2.83, *p* = 0.042). Neither the HSB nor the LSB patients showed significant changes in IFN activity at 24 weeks. There were no significant differences in ESSPRI in the PDF (*n* = 39) and DDF (*n* = 22) patients taking HCQ. However, significant reductions in overall IFN score at 24 weeks were seen in both PDF (difference at 24 weeks; 6.41, 95% CI, 2.48–10.34, *p* = 0.002) and DDF (difference at 24 weeks; 7.23, 95% CI, 1.85–12.6, *p* = 0.009) without improvement in ESSPRI. Although the JOQUER trial reported no overall benefit from HCQ in pSS patients, stratification suggests that both HSB and LSB subgroups may respond to HCQ. However, these patients may benefit through mechanisms other than the reduction of IFN activities.

## Introduction

Hydroxychloroquine (HCQ) is an immunomodulatory drug widely prescribed for primary Sjögren’s syndrome (pSS). The mechanism of action of HCQ in pSS is not fully understood but is believed to mediate through interference with antigen presentation by altering lysosomal pH and inhibition of toll-like receptor signalling [1]. In turn, this may down-regulate interferon activation [2].

The effectiveness of HCQ in treating pSS remains under debate. The JOQUER trial tested 120 patients with pSS in a randomised controlled trial (RCT) in which patients were randomized (1:1) to receive HCQ (400 mg OD) or placebo from baseline until 24 weeks [3]. Between weeks 24 and 48, all participants were prescribed HCQ. In addition, the interferon-stimulated genes IF144, IF144L, IFIT1, IFIT3 and MX-1 and an IFN score—defined by the sum of the gene expression values for the five interferon-stimulated genes (ISGs)—were evaluated at baseline and 24 weeks [4]. Results from this trial showed a statistically significant down-regulation of ISGs and IFN score, but no overall clinical benefit compared to placebo.

pSS is a very heterogenous disorder and Tarn et al*.* (2019) identified four Sjögren’s syndrome subgroups based on patient-reported symptoms [5]. These included the high-symptom burden (HSB), pain dominant with fatigue (PDF), dryness dominant with fatigue (DDF) and low symptom burden (LSB), with each subgroup having distinct pathobiologies underpinned by differences in transcriptomic profiles and IFN modular activities. We hypothesize that these subgroups may display a differential response to HCQ. Preliminary analysis using stratified data from the JOQUER trial demonstrated that the HSB group showed improvement in ESSPRI in response to HCQ compared with placebo [5]. However, since HSB patients demonstrate higher ESSPRI scores at baseline, the positive response to HCQ seen in this group could be considered “regression to the mean”. Therefore, it is of interest to re-evaluate in further detail adjusting for baseline differences, stratifying by subgroup and exploring the differential interferon response to HCQ compared to placebo.

## Methods

### Design

The JOQUER trial was approved by the institutional review board of Hôpital Bichat (Paris, France). The study was conducted according to the principles of the Declaration of Helsinki. Informed consent was obtained from all patients. We obtained data from the JOQUER trial for re-analysis.

### Patient group

We included 107 patients from the JOQUER trial with patient-reported symptoms at baseline permitting stratification into four distinct subgroups (HSB, DDF, PDF and LSB) based on the NSST method as previously described [5]. In brief, clusters of patients were identified in relation to five primary pSS symptoms including pain, fatigue, dryness, anxiety and depression. The HSB subgroup includes patients that have high scores from all five symptoms whereas the LSB subgroup patients score low on all symptoms. The PDF patients have high pain and fatigue scores and the DDF subgroup score high for dryness and fatigue. Both PDF and DDF subgroups score low on anxiety and depression scores. Sixty-eight of those patients with IFN-related data available. All patients fulfilled the American-European Consensus Group Criteria for pSS.

### Outcomes

A comprehensive analysis of primary and secondary outcomes was tested in the JOQUER trial and performed after stratifying the patients. These included individual symptoms (pain, fatigue, dryness, anxiety and depression), location of dryness, EULAR Sjögren’s syndrome patient reported index (ESSPRI), EULAR Sjögren’s syndrome disease activity index (ESSDAI), Profile of Fatigue (ProF) [6], sicca symptoms inventory (SSI), Schirmer’s test and unstimulated salivary flow (USF). In addition, changes in the IFN score measured as a weighted combination of the gene expression of five IFN-stimulated genes IF144, IF144L, IFIT1, IFIT3 and MX-1 relative to age and sex-matched health controls was analysed [4, 7].

### Data analysis

To make the best use of the available data, we used a repeated-measures moving covariates model—a class of transition model for the analysis of longitudinal data, [8, 9] exploiting data in earlier time points (baseline or 12 weeks) as a covariate in the model. In addition, the model included drug treatment, subgroup, and their interaction, followed by contrasts to compare drug treatments within each subgroup [10, 11]. Data at baseline, 12 and 24 weeks were analyzed using the statistical package SAS JMP Pro Version 13 and supplementary analyses were performed using the SAS MIXED Procedure fitting a mixed-effects model. For a range of alternative error structures, these analyses confirmed the findings of the simpler covariance analyses and are not reported further. Week 48 data collected following unblinding at week 24 and switching of placebo to HCQ were excluded from the analysis.

## Results

**Fig. 1:**
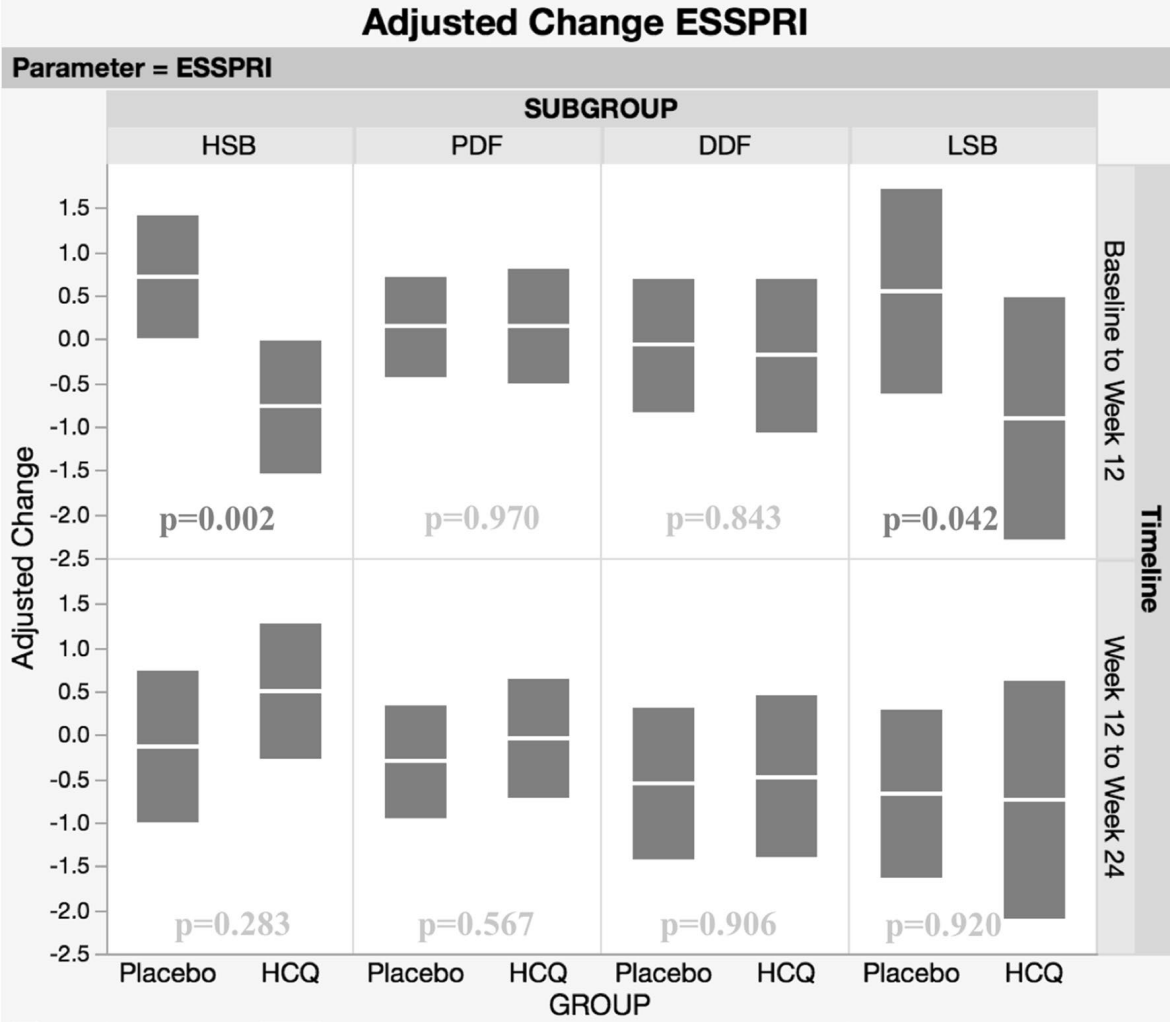
Adjusted changes and 95% confidence limits for ESSPRI between Baseline and Week 12, Week 12 and Week 24. Footnote: sample sizes for both ESSPRI and ESSDAI. Scores were available for 14 LSB patients (9 Placebo, 5 HCQ), 32 HSB patients (18 Placebo, 14 HCQ), 22 DDF patients (11 Placebo, 11 HCQ) and 39 PDF patients (20 Placebo, 19 HCQ)

**Fig. 2:**
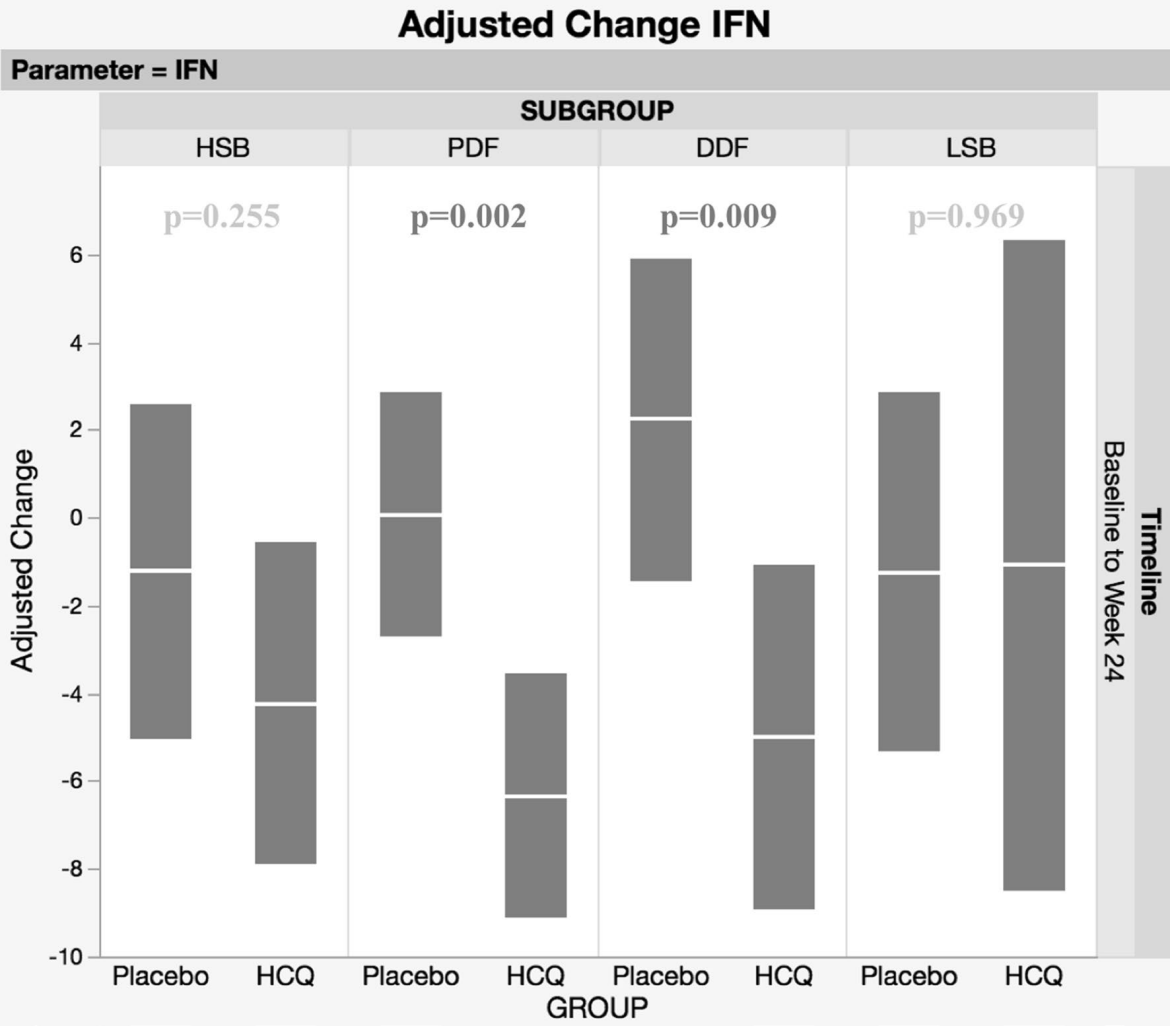
Adjusted changes and 95% confidence limits for the IFN Score between Baseline and Week 24 following adjustment for Baseline values. There are reductions in IFN scores in both the PDF and DDF subgroups for patients treated with HCQ. IFN scores were analysed for 9 LSB patients (7 Placebo, 2 HCQ), 16 HSB patients (8 Placebo, 8 HCQ), 15 DDF patients (8 Placebo, 7 HCQ) and 28 PDF patients (14 Placebo, 14 HCQ)

**Fig. 3:**
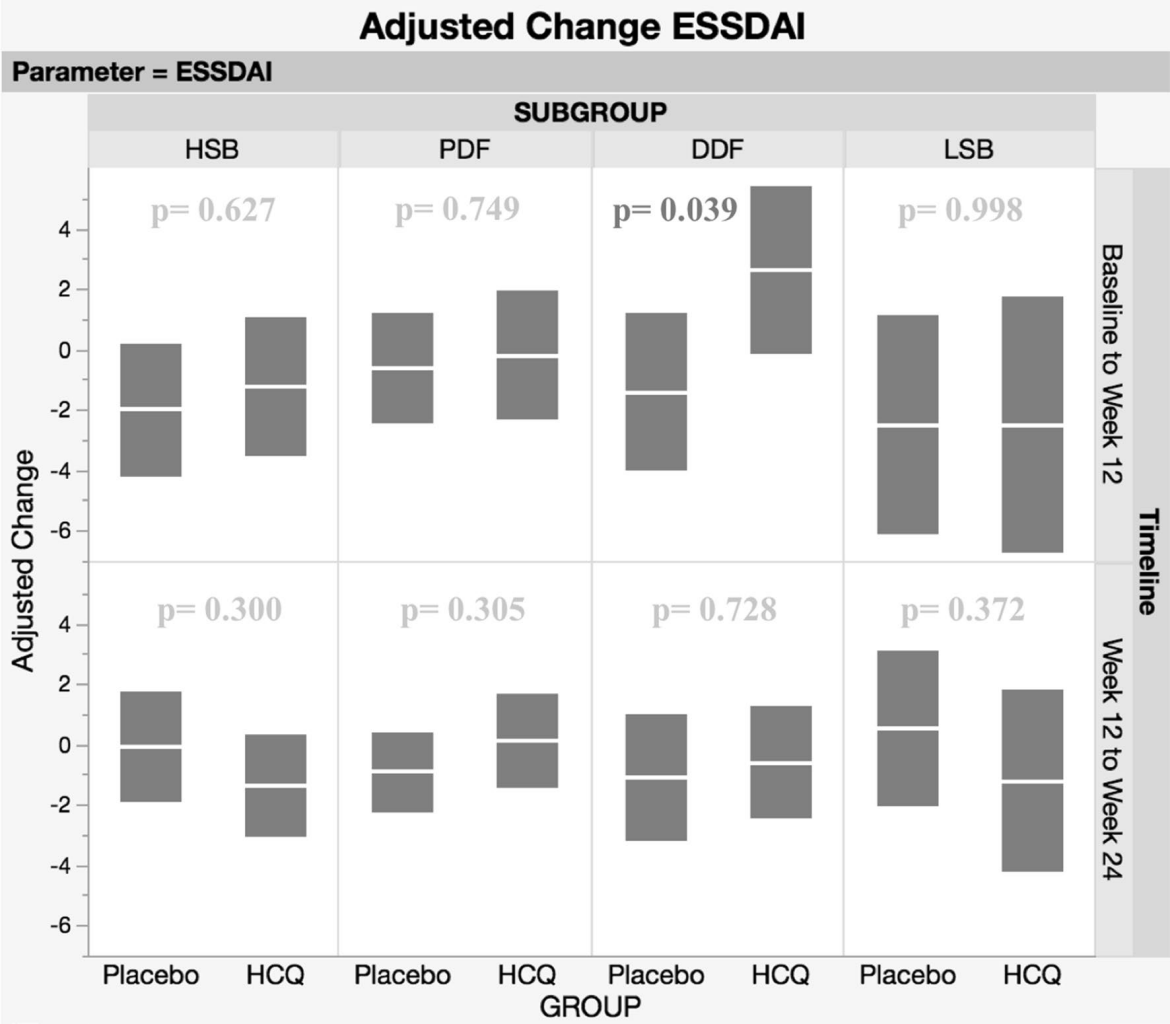
Adjusted changes and 95% confidence limits for ESSDAI between Baseline and Week 12, Week 12 and Week 24

Of the 107 patients stratified at baseline, 32 patients were classified as HSB (18 Placebo, 14 HCQ), 39 as PDF (20 Placebo, 19 HCQ), 22 as DDF (11 Placebo, 11 HCQ), and 14 patients were classified as LSB patients (9 Placebo, 5 HCQ). IFN scores were available for 16 HSB patients (8 Placebo, 8 HCQ), 28 PDF patients (14 Placebo, 14 HCQ), 15 DDF patients (8 Placebo, 7 HCQ) and 9 LSB patients (7 Placebo, 2 HCQ). Figure 1 shows adjusted changes and 95% confidence limits for these changes in ESSPRI. Figure 2 for IFN scores at relevant time points. Figure 3 shows adjusted changes in ESSDAI. Summary statistics (medians and quartile ranges) are presented for the relevant variables in Table 1.

**Table 1:** Medians and quartiles (LQ-UQ) for variables for the High Symptom Burden (HSB), Low Symptom Burden (LSB), Dryness Dominant Fatigue (DDF), and Pain Dominant Fatigue (PDF) subgroups at baseline, week 12 and/or week 24 of treatment with Placebo or Hydroxycholoroquine (HCQ)

Parameter	Week	HSB				DDF				PDF				LSB			
		Placebo		HCQ		Placebo		HCQ		Placebo		HCQ		Placebo		HCQ	
		Median	(LQ, UQ)	Median	(LQ, UQ)	Median	(LQ, UQ)	Median	(LQ, UQ)	Median	(LQ, UQ)	Median	(LQ, UQ)	Median	(LQ, UQ)	Median	(LQ, UQ)
ESSPRI	0	7.17	(6.3, 8)	7.33	(6.8, 7.9)	5	(4.7, 5.7)	4.33	(4, 5.3)	6.83	(5.4, 7.7)	6.67	(5.7, 7.3)	2.67	(1.8, 2.8)	2.33	(1.5, 3.2)
	12	7.67	(6.7, 8.3)	6.33^a^	(5.5, 7.2)	5.33	(4.7, 6.3)	5	(3.8, 6.3)	6.33	(5.8, 7.6)	7	(5.7, 8.2)	4.67	(2.5, 5)	2.67^a^	(1.5, 3.8)
	24	7.00	(5.7, 8)	7.17	(4.7, 7.3)	5.5	(3.8, 6.7)	4.83	(3.3, 5.9)	6.33	(4.3, 7.3)	6.5	(5, 7.33)	5	(1.7, 5.7)	2.67	(2, 3.8)
Pain	0	7.5	(6.8, 8)	7	(5.8, 9)	1	(1, 3)	2	(0, 3)	7	(5.3, 8)	7	(6, 8.8)	1	(0, 2)	2	(0, 2)
	12	7	(6, 8)	6	(4, 6.5)	3	(1, 6)	4^a^	(0, 5)	6	(4.25, 8)	7	(5.5, 8)	3	(1.5, 5)	2	(0, 3)
	24	6	(5, 8)	5.5	(4.3, 7.8)	3	(1.5, 4.3)	2	(0, 5)	7	(5, 8)	6	(5, 8)	4	(1.5, 6)	1	(0.5, 2.5)
Dryness	0	7	(5.8, 8)	8	(7.8, 8.3)	7	(6.5, 9.5)	8	(7.5, 8)	7	(6, 8)	6	(5, 7)	3	(2, 4)	4	(2, 6)
	12	7.5	(7, 9.8)	7^a^	(4, 8)	7	(6, 10)	7.5	(6, 8.8)	7.5	(6, 8)	7.5	(5, 8.8)	5	(1, 6)	4	(2, 5)
	24	7	(5, 9)	7	(5, 8)	5.5	(4.3, 9)	7	(5.5, 8)	6	(4, 8)	6	(5.5, 7.5)	4	(1.5, 6.5)	3	(2.5,7.5)
Fatigue	0	7	(6.75, 8)	8	(6, 8.3)	7	(4, 8)	4.5	(3.3, 5.8)	7	(5, 9)	7	(7, 9)	3	(1.5, 3.5)	2	(0.5, 3)
	12	7	(6, 8)	6^a^	(4, 6.5)	3	(1, 6)	4	(0, 5)	6	(4.3, 8)	7	(5.5, 8)	3	(1.5, 5)	2	(0, 3)
	24	6	(5, 8)	5.5	(4.3, 7.8)	3	(1.5, 4.3)	2	(0, 5)	7	(5, 8)	6	(5, 8)	4	(1.5, 6)	1	(0.5, 2.5)
ESSDAI	0	2	(2, 8)	2	(0, 5.3)	3	(0, 6)	6	(0, 10)	2	(0.3, 6.8)	2	(1.3, 5)	3	(2, 5.5)	0	(0, 3)
	12	2	(0, 4)	1.5	(0, 2.75)	1.5	(0, 3.5)	7^a^	(0, 12)	2	(0, 6.5)	3.5	(0.5, 5.8)	2	(0.5, 3.5)	0	(0, 2)
	24	3	(2, 7)	1.5	(0, 5)	2	(0, 5.5)	2.5	(0, 6.3)	2	(0, 5)	2	(0.5, 2.5)	4	(2, 6)	0	(0, 2)
Schirmer’s	0	4.3	(2, 7.9)	12	(1.9, 16.3)	2	(1.1, 4.5)	7.8	(0, 9.1)	6.5	(3.5, 17.5)	6	(4, 15)	11.8	(2.5, 24.3)	20	(0.75, 26.5)
	24	5.0	(2, 7.5)	14.5^a^	(7.3, 20.5)	5	(1.5, 17)	6.5	(1, 8)	3.5	(1.5, 9.3)	5.8	(5, 13.9)	6.8	(3.3, 21.6)	6.3	(5.3, 21.1)
USF	0	0.04	(0, 0.2)	0.2	(0, 0.34)	0.3	(0.1, 0.3)	0.09	(0.04, 0.1)	0.08	(0.01, 0.1)	0.1	(0.09, 0.2)	0.3	(0.1, 0.5)	0.06	(0.03, 0.2)
	24	0.02	(0, 0.2)	0.1	(0.02, 0.2)	0.1	(0, 0.3)	0.1	(0.1, 0.7)	0.1	(0.03, 0.1)	0.1	(0.08, 0.3)	0.34	(0.1, 0.7)	0.3^a^	(0.04, 0.3)
PoF	0	6.0	(5.5, 7.2)	5.7	(3.9, 6.6)	3.6	(1.7, 4.2)	3.4	(1.1, 4.6)	5.3	(3.7, 6.5)	5.9	(4.3, 8.1)	2.2	(1.2, 3.3)	2.1	(1.3, 3.5)
	24	7.0	(5.6, 7.9)	6.2	(4.3, 6.6)	3.1	(1, 4.3)	4.0	(1.4, 5.3)	4.9	(2.2, 6.7)	5.3	(3.6, 7.1)	3.8	(3.1, 6.1)	2^a^	(1.5, 2.7)
IFN score	0	2.6	(− 4.3, 7.8)	8.9	(−1.7, 18.4)	12.2	(0.2, 17.9)	5.5	(0.6, 16.5)	9.7	(3.8, 15.2)	8.5	(−2.3, 15.1)	17.7	(13.8, 18.6)	15.1	(12.4, 17.7)
	24	0.3	(− 4.9, 8.4)	3.4	(−4.5, 12.5)	13.6	(8.6, 17.8)	−0.01^a^	(− 5, 13.5)	10.9	(0.5, 15.2)	0.3^a^	(−5.4, 6.8)	16.8	(9.6, 19)	12.8	(11.8, 13.9)

^**a**^Denotes statistically significant changes between HCQ and placebo group from baseline. Schirmer’s test, unstimulated salivary flow (USF), Profile of fatigue (PoF) and IFN score were only measured at baseline and week 24, therefore, no data at week 12 was available

### HSB

Adjusting for differences at baseline confirms the observation reported by Tarn et al. of statistically significant improvements in ESSPRI in the HSB group for HCQ-treated patients compared to placebo controls. (5) By 12 weeks, patients treated with HCQ show an adjusted difference in ESSPRI compared to placebo of 1.49 points (95% CI 0.54–2.43; *p* = 0.002). There was no further statistically significant adjusted change in ESSPRI for HSB patients between week 12 and week 24. Improvement was seen in all three ESSPRI sub-scores—pain, fatigue and dryness (Table 1). There was no significant adjusted change in ESSDAI (difference at 12 weeks: 0.78, 95% CI, − 2.41–3.97, *p* = 0.627) and no further change at 24 weeks for the HSB groups, nor were there significant changes in the IFN scores (difference at 24 weeks: 3.03, 95% CI, − 2.25–8.30, *p* = 0.255; IFN score was not measured at week 12 in this trial).

### PDF

There were no significant clinical differences observed in the PDF group, including ESSPRI (and individual components of ESSPRI) and ESSDAI. However, all five ISGs were significantly down-regulated in the HCQ group, with a statistically significant adjusted decrease in the overall IFN score (difference at 24 weeks; 6.41, 95% CI, 2.48–10.34, *p* = 0.002) compared to placebo.

### DDF

There were no statistically significant differences in ESSPRI in DDF patients. However, after 12 weeks, there was a small increase in the ESSDAI scores in the HCQ group and a decrease in the placebo group (difference at 12 weeks; 4.04, 95% CI, 0.21–7.86, *p* = 0.039) but no further statistical difference at 24 weeks (difference at 24 weeks; 0.50 95% CI, − 2.34–3.33, *p* = 0.728). Interestingly, comparing treatment groups, IFN score decreased after 24 weeks (difference at 24 weeks; 7.23, 95% CI, 1.85–12.6, *p* = 0.009) in the HCQ group.

### LSB

By 12 weeks, an adjusted difference of 1.44 points in the ESSPRI scores between LSB patients randomised to HCQ and placebo (95% CI, 0.05–2.83, *p* = 0.042) was observed. The difference was due to a rise in ESSPRI score in the placebo group. Changes in ESSPRI arose largely from changes in pain and dryness (Table 1). There was no further statistically significant change in ESSPRI between week 12 and week 24. Profile of Fatigue scores increased in the placebo group whereas there was a very mild decrease in the HCQ group (difference at 24 weeks, 2.18; 95% CI, 0.49–3.86, *p* = 0.012) and USF (difference at 24 weeks; 0.34; 95% CI, 0.04–0.07, *p* = 0.030) all improved with HCQ compared to placebo after 24 weeks. There were no significant differences in ESSDAI or the IFN score. The number of LSB patients in this trial was smaller than the other groups and caution for the interpretation of these findings is needed.

## Discussion

Re-analysing the trial data, adjusting for baseline differences and stratifying into the four subgroups reported by Tarn et al. [5] suggests that patients in the HSB subgroup demonstrate clinically meaningful improvements from taking HCQ with reductions in ESSPRI and all three subscores of pain, fatigue and dryness.

Using unstratified data of the JOQUER trial, Bodewes et al. showed reduced IFN scores and ISGs in the HCQ group compared to placebo [4]. Our analysis showed that ISG levels and IFN scores were downregulated by HCQ predominantly in the PDF and DDF groups, but with no improvement in clinical responses. Paradoxically, while the HSB and LSB groups showed clinical response to HCQ there were no significant changes in the ISG levels or IFN scores. Our findings suggest that there is a dissociation between the improvement in IFN signatures and clinical status in each of the pSS subgroups. Furthermore, the differential effects of HCQ on IFN scores between the four sub-groups reinforce the concept that these are distinct endotypes. It should be noted that biological samples were not available at 12 weeks for the measurement of ISG levels and IFN scores.

Our data is consistent with the recent reports that increased fatigue scores are associated with lower serum levels of proinflammatory cytokines [7, 12, 13], and that improvement in fatigue in response to a nuclease therapy was associated with an increase in IFN modular activity in pSS patients [14]. Taken together, our findings challenge the presumed mechanisms of action of HCQ [15] and prompt further investigations into the role of IFN activity in pSS pathobiology. Our findings also underscore the clinical and biological significance of the NSST pSS subgroups.

There are limitations to this study. While it is true that component scores of ESSPRI at baseline are used for the symptom-based stratification and final ESSPRI is used as the main clinical outcome, our re-analysis was based on comparison to the placebo arm, adjusted for those baseline ESSPRI scores, and is unlikely to be attributable simply to bias inherent to the stratification approach or “regression to the mean”. The original JOQUER trial was not powered for stratification by subgroups and there were imbalances in the number of patients in each subgroup and treatment group. For example, LSB patients are less likely to be recruited to trials and indeed make up the smallest subgroup in this study. Incomplete data meant we were unable to stratify 13 (10.8%) of the trial cohort. Non-random missingness could conceivably give rise to biases in the data. In addition, samples were not available for interferon analysis at 12 weeks. For these reasons, we urge caution in the interpretation of our results. We encourage adequately powered randomized clinical trials of HCQ with stratification of patients and sample size calculations to better estimate treatment effects in each subgroup. This study highlights that HCQ may reduce the overall symptom burden in specific patient subgroups. During the study, those patients showing down-regulation of IFN pathways in response to HCQ did not improve clinically. This has implications for the treatment and care pathway of pSS patients presenting in the clinic.

## Data Availability

Relevant data and material may be available to share upon request.
